# The complete chloroplast genome sequence of *Tussilago farfara* (Asteraceae)

**DOI:** 10.1080/23802359.2021.2005494

**Published:** 2022-03-25

**Authors:** Duan Yizhong, Ke Lu, Du Zhongyu, Yehua Shen

**Affiliations:** a College of Chemistry and Materials Science, Northwest University, Xi'an, China; b Shaanxi Key Laboratory of Ecological Restoration in Northern Shaanxi Mining Area, Yulin University, Yulin, China; c Breeding Base for State Key Laboratory of Land Degradation and Ecological Restoration in Northwest China, Ningxia University, Yinchuan, China

**Keywords:** *Tussilago farfara*, complete chloroplast genome, phylogenetic analysis

## Abstract

*Tussilago farfara* is a member of the family Asteraceae. In this paper, we reported the complete chloroplast (cp) genome sequence of *T. farfara*. The results showed that *T. farfara* complete chloroplast genome comprises 151,325 bp, containing a largen single copy (LSC) region of 83,370 bp, a small single copy (SSC) region of 18,273 bp, and a pair of inverted repeats (IRs) region of 24,841 bp. The genome has a GC content of 37.4%. The LSC, SSC, and IR regions represent 35.5%, 30.6%, and 43.0% of the *T. farfara* chloroplast genome length. We annotated 132 genes, comprising 87 protein-coding genes (PCGs), eight rRNA genes, and 36 tRNA genes. A phylogenetic analysis based on 31 cpDNA genomes suggested that the *T. farfara* is closely related to *Farfugium japonicum*.

*Tussilago farfara* (Linaeus 1753) belongs to the genus *Tussilago* of the family Asteraceae, it is an important medicinal material, flower buds and leaves are used as medicine to relieve cough, moisten lung and reduce phlegm (Song et al. 2021). It is also a honey sea plant, which is widely distributed in medicinal nurseries, valleys, wetlands, and forests all over China (Fedina et al. 2020). Complete chloroplast (cp) genome is the theoretical basis and experimental material for studying speciation, genetic diversity of germplasm resources, and gene engineering technology of higher plants (Lu et al. 2020). At present, it mainly focuses on physiological and biochemical aspects (Song et al. 2020). In this study, we reported the cpDNA genome sequences of *T. farfara.* We hope to provide a theoretical basis for the chloroplast genome characteristics and the phylogenetic relationship of this species.

The fresh leaves of *T. farfara* were provided from Yulin Forestry Industry Center, Shaanxi, China (109°44′20.31″E, 38°14′58.58″N) in July 2021. The specimens of *T. farfara* (Accession Number: 20210910Yl02) were deposited at the Herbarium of Yulin University (https://www.yulinu.edu.cn/, Duan Yizhong, duanyizhong2006@163.com), Shaanxi, China. Total DNA was extracted from the fresh leaves according to a modified cetyltrimethyl ammonium bromide (CTAB) method (Doyle and Doyle 1987). The subsequent high-throughput sequencing was completed with the IIIuminaHiSeq Ten system. The *Farfugium japonicum* (MT929248) cpDNA genome was used as the reference sequence to annotate. The cpDNA genome of *T. farfara* was annotated using the Geneious 8.0.2 (Kearse et al. 2012). The physical map of *T. farfara* was visualized by OGDRAW online tool (Lohse et al. 2013). We aligned all 31 cpDNA genome sequences used MAFFT (Kazutaka et al. 2002) and the MEGA 7.0 program (Kumar et al. 2016) to construct a phylogenetic tree according to the Neighbour-joining (NJ) method, with a bootstrap value of 1000. The annotated *T. farfara* cpDNA genome sequence has been deposited into the GenBank database (accession number: MW760850).

Our result showed that complete chloroplast genome of *T. farfara* is 151,325 bp, similar with the chloroplast structure of most other plants (Shinozaki et al. 1986), with a typical quadripartite structure containing a large single copy (LSC) region of 83,370 bp, a small single copy (SSC) region of 18,273 bp, and a pair of inverted repeats (IRs) region of 24,841 bp. The genome has a GC content of 37.4%. The LSC, SSC, and IR regions represent 35.5%、30.6% and 43.0% of the *T. farfara.* We annotated 132 genes, including 87 protein-coding genes, 8rRNAs, and 36 tRNAs. We constructed a phylogenetic tree based on 31 cpDNA genome sequences, with the closely related two species of *Nymphoides*, *Nymphoides coreana* (NC 041481) and *Nymphoides coronate* (NC 041484) cpDNA genome serving as an outgroup (Figure 1). The result showed that the *T. farfara* (MW760850) is closely related to *Farfugium japonicum* (MT929248).

**Figure 1.**
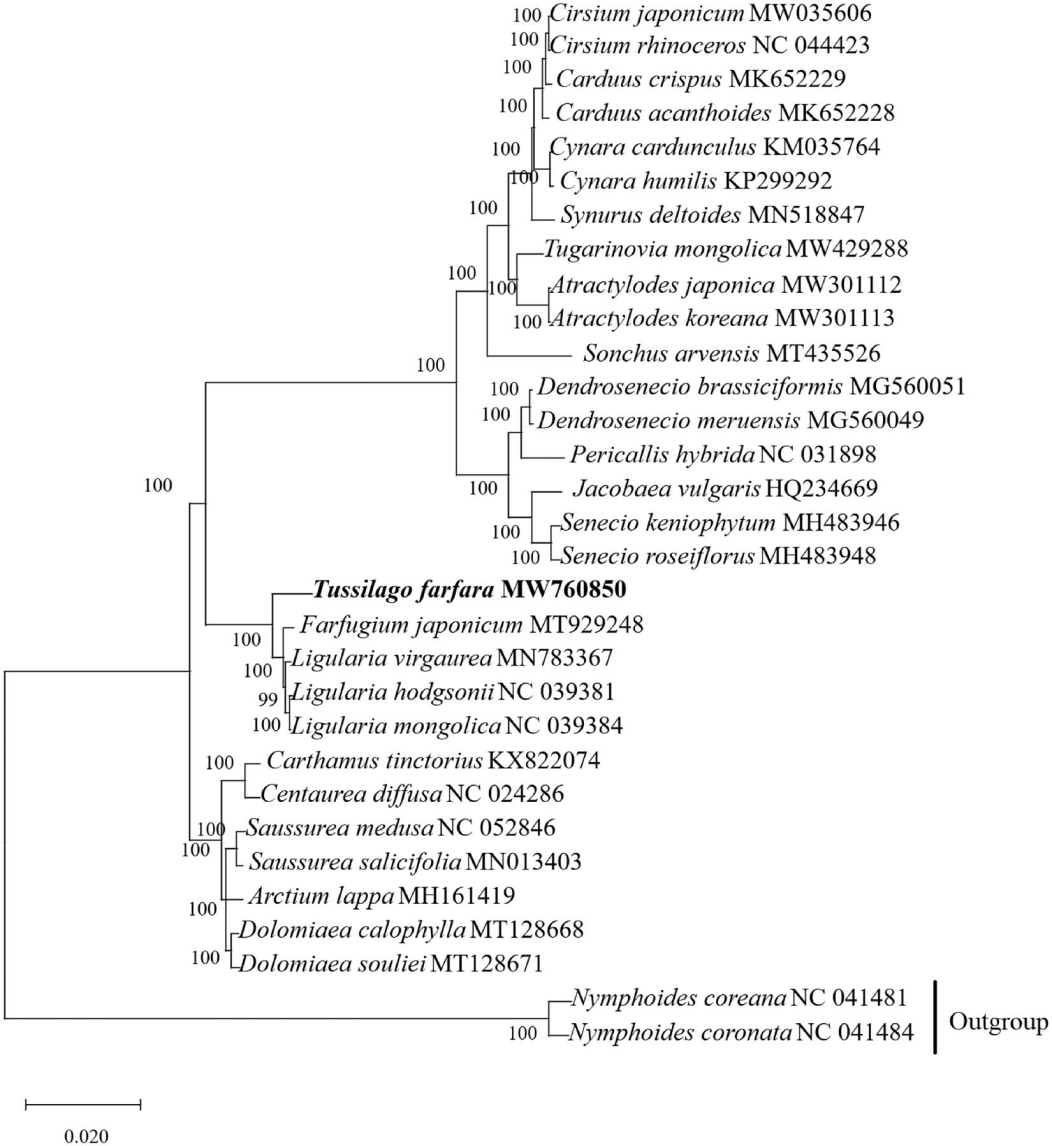
Phylogenetic tree constructed based on 31 species of chloroplast genome sequences. Accession numbers*: Cirsium japonicum* (MW 035606); *Cirsium rhinoceros* (NC 044423); *Cardus crispus* (MK652229); *Cardms acanthoides* (MK652228); *Cynara cardunculus* (KM035764*)*; *Cynara humilis* (KP299292); *Symrus deltoides* (MN518847)*; Tugarinovia mongolica* (MW429288); *Atractylodes japonica* (MW301112); *Atractylodes koreana* (MW301113); *Sonchus arvensis* (MT435526); *Dendrosenecio brassiciformis* (MG560051); *Dendrosenecio meruensis* (MG560049*)*; *Pericallis hybrida* (NC 031898); *Jacobaea vulgaris* (HQ234669); *Senecio keniophym* (MH483946); *Senecio roseiflorus* (MH483948); *Tussilago farfara* (MW760850); *Farfugium japonicum* (MT929248); *Ligularia virgaurea* (MN783367); *Ligularia hodgsonii* (NC 039381); *Ligularia mongolica* (NC 039384); *Carthamus tinctorius* (KX822074); *Centarea diffusa* (NC 024286); *Saussurea medusa* (NC 052846); *Saussurea salicifolia* (MN013403); *Arctium lappa* (MH161419); *Dolomiaea calophlla* (MT128668); *Dolomiaea souliei* (MT128671); *Nymphoides coreana* (NC 041481); *Nymphoides coronate* (NC 041484).

## Data Availability

The genome sequence data that support the findings of this study are openly available in GenBank of NCBI at [https://www.ncbi.nlm.nih.gov] (https://www.ncbi.nlm.nih.gov/) under the accession no. MW760850. The associated BioProject, SRA, and Bio-Sample numbers are PRJNA765479, SRR16017860, and SAMN21561337 respectively.
